# Glymphatic dysfunction in amyotrophic lateral sclerosis: a multimodal MRI investigation of brain-CSF functional and structural dynamics

**DOI:** 10.3389/fnins.2025.1666114

**Published:** 2025-10-06

**Authors:** Zelin Liu, Hui Dong, Haiqing Yang, Lixia Zhou, Min Li, Xinyi Zhang, Yuanhui Zhao, Meiqi Han, Yaling Liu, Zuojun Geng

**Affiliations:** ^1^Department of Radiology, The Second Hospital of Hebei Medical University, Shijiazhuang, Hebei, China; ^2^Department of Neurology, The Second Hospital of Hebei Medical University, Shijiazhuang, Hebei, China; ^3^Affiliated Hospital of Chengde Medical University, Chengde, Hebei, China; ^4^Key Laboratory of Clinical Neurology, Ministry of Education, Hebei Medical University, Shijiazhuang, Hebei, China; ^5^Key Neurological Laboratory of Hebei Province, Shijiazhuang, Hebei, China

**Keywords:** amyotrophic lateral sclerosis, glymphatic system, gBOLD-CSF coupling, DTI-ALPS, choroid plexus volume

## Abstract

**Background:**

Amyotrophic lateral sclerosis (ALS) is characterized by progressive motor neuron degeneration and glial activation. The coupling of global blood oxygen level-dependent (gBOLD) signals with cerebrospinal fluid (CSF) inflow dynamics is a novel non-invasive biomarker, which is applied to assess the relationship between lymphatic function and ALS.

**Objective:**

The gBOLD-CSF coupling was used to assess the glymphatic system dysfunction related to ALS, and the relationship between this disease and the glymphatic system was further explored by combining the diffusion tensor imaging index of the perivascular space (DTI-ALPS) and the volume fraction of the choroid plexus (choroid plexus volume [CPV]/intracranial total volume [TIV]).

**Methods:**

We conducted a systematic analysis and comparative study of the imaging indicators and clinical data of 41 patients with ALS and 43 healthy controls (HC).

**Results:**

ALS patients showed significantly reduced gBOLD-CSF coupling (*p* < 0.001), reduced ALPS index (*p* < 0.001), and increased CPV fraction (*p* < 0.001). The area under the ROC curve (AUC) were 0.790 (gBOLD-CSF), 0.760 (ALPS index), and 0.748 (CPV fraction). A diagnostic model for ALS was developed based on gBOLD-CSF coupling, ALPS index, and CPV fraction with an AUC of 0.897 (0.830–0.964). The calibration curve demonstrates that the model exhibits strong consistency. The results of the Decision Curve Analysis (DCA) further indicate that the nomogram possesses substantial clinical utility.

**Conclusion:**

This study identified that gBOLD-CSF coupling has diagnostic value for ALS and developed a diagnostic model by combining the ALPS index and CPV fraction, which has good diagnostic efficacy and clinical application value.

## 1 Introduction

Amyotrophic lateral sclerosis (ALS) is a complicated neurological condition marked by varying clinical presentations and diverse genetic underpinnings, characterized by swift disease advancement and the deterioration of motor neurons, with most patients surviving around 30 months after symptoms first appear. Patients experience progressive muscle weakness, which typically results in paralysis and ultimately leads to fatal respiratory failure (Taylor et al., 2016). The processes that contribute to the onset and progression of ALS involve complex mechanisms, and current therapies show limited efficacy. Additionally, the absence of reliable molecular markers makes it difficult to effectively monitor disease progression and assess treatment effectiveness (Turner and Benatar, 2015). Currently, the Revised Amyotrophic Lateral Sclerosis Functional Rating Scale (ALSFRS-R) is primarily used in clinical practice as an outcome indicator for evaluating the condition of patients and validating the efficacy of therapeutic trials. However, this scale has certain limitations in practical application (Proudfoot et al., 2016). High-field-strength magnetic resonance imaging (MRI) technology’s functional and structural sequences can visualize the abnormal structures and functions in central nervous system (CNS) diseases. MRI is anticipated to offer valuable insights for assessing disease conditions, including monitoring disease advancement and assessing therapeutic effectiveness (Verstraete and Turner, 2015).

The glymphatic system and meningeal lymphatic vessels play a critical role in maintaining central nervous system (CNS) homeostasis (Eisen et al., 2024). The glymphatic system appears essential for delaying or preventing neurodegenerative processes and disease-associated clinical disability in patients with amyotrophic lateral sclerosis (ALS; Kwong et al., 2020). Impairment of this system could intensify neuroinflammatory responses and contribute to the buildup of abnormally folded proteins, impairing the clearance of neuro-damaging proteins like TDP-43 (Boland et al., 2018). In the past, most studies on the glymphatic system of the human brain have employed dynamic contrast enhanced (DCE) MRI technology, which involves intrathecal or intravenous administration of contrast media. As an invasive operation method, its repeated application may lead to abnormal accumulation of gadolinium agents in brain tissue. Meanwhile, this protocol has clear contraindications for patients with impaired liver and kidney functions, which restricts its clinical application (Gulani et al., 2017). In recent years, several crucial biomarkers for glymphatic imaging in various neurological disorders have emerged, such as gBOLD-CSF coupling, ALPS-index, and CPV score (Bae et al., 2023; Han et al., 2021; Liu et al., 2023; Choi et al., 2025).

Perivascular space analysis via diffusion tensor imaging (DTI-ALPS) offers an innovative imaging approach for non-invasive evaluation of the lymphatic drainage function of the human central nervous system (Taoka et al., 2017). In addition, a notable correlation exists between the evaluation results obtained indirectly through the DTI-ALPS method for measuring lymphatic function and those obtained directly by intrathecal injection of neural tracers (Zhang et al., 2021). Previous studies have established the practical application value of DTI-ALPS in diverse CNS illnesses, including Alzheimer’s disease dementia (AD; Taoka et al., 2017), Parkinson’s disease (PD; Mcknight et al., 2021), and ALS (Huang et al., 2025).

MRI studies have shown that the function of the glymphatic system in ALS patients is significantly impaired. The ALPS index shows a highly significant negative correlation with the CPV fraction (Choi et al., 2025). The DTI-ALPS index decreased in patients with ALS, and longitudinal observation showed a significant downward trend with age (Sharkey et al., 2024). A significant inverse relationship between ALSFRS-R scores and CPV in ALS (Dai et al., 2024), There is not many studies on the two indicators, DTI-ALPS and CPV, in ALS research. The relationship between them still needs further verification, and the DTI-ALPS and CPV scores cannot directly reflect the dynamic aspects of the lymphatic system.

Global blood oxygen level-dependent (gBOLD) resting-state functional MRI (rs-fMRI) signals at frequencies below 0.1 Hz are associated with cerebrospinal fluid (CSF) dynamics. This low-frequency component is present in many brain regions and increases significantly during sleep (Fukunaga et al., 2006), surpassing pulsations related to heart rate and breathing (Helakari et al., 2022). Research shows that the average low-frequency rs-fMRI signal in all gray matter regions (gBOLD) is closely related to fourth ventricle fluctuations during sleep (Picchioni et al., 2022; Fultz et al., 2019). This relationship is especially clear in isolated events, where the sharp drop in gBOLD aligns with the cerebrospinal fluid peak (Fultz et al., 2019). Although gBOLD and CSF signals both come from fMRI, they originate in different brain regions and involve distinct mechanisms: gBOLD reflects blood oxygen changes in gray matter (Ogawa et al., 1990), while CSF signals arise from untagged CSF inflow outside the imaging area, known as the MRI inflow effect (Gao and Liu, 2012). It is worth noting that spontaneous gBOLD and cerebrospinal fluid fluctuations align with slow changes in neural and physiological signals like electroencephalogram, heart rate, and respiration (Gu et al., 2022). In summary,this gBOLD-CSF coupling reflects CSF movement (Fultz et al., 2019). The coupling of gBOLD and CSF signals, defined as a peak negative correlation at a specific time lag, serves as an important indicator of lymphatic function, reflecting coordination between neuronal activity and CSF flow, and offering a new non-invasive method to assess brain waste clearance (Wang et al., 2023). The diminished strength of gBOLD-CSF coupling has been observed in various CNS illnesses, including PD (Wang et al., 2023), AD (Han et al., 2021) and moyamoya disease (Zhu et al., 2024). However, the precise alterations of this parameter in amyotrophic lateral sclerosis (ALS) have yet to be clarified.

Therefore, this study employed the gBOLD-CSF coupling method to assess the glymphatic system dysfunction associated with ALS, and further explored the potential association between ALS and the glymphatic system by integrating multiple imaging metrics such as DTI-ALPS and CPV fraction.

## 2 Materials and methods

### 2.1 Study population

We continuously recruit patients diagnosed with ALS according to the Gold Coast criteria (Hannaford et al., 2021), who were enrolled from Hebei Medical University’s Second Affiliated Hospital, Shijiazhuang, China between March 2024 and April 2025. During the research period, this study rigorously adhered to the matching principle. Healthy Controls (HC) were recruited using identical 3.0 T magnetic resonance imaging equipment at the same medical institution. These healthy controls exhibited no statistically significant differences from the disease group concerning gender, age, body mass index (BMI), and education. The HC group was selected based on the following, ① No abnormal signs were detected through standardized neurological examinations; ② No evident abnormal signals were identified in routine brain magnetic resonance imaging (MRI) assessments. This research received ethical approval from the Ethics Review Committee at Hebei Medical University’s Second Hospital (Approval No. 2024-R193), and all participants provided written informed consent prior to their involvement in the study.

### 2.2 Clinical parameters of ALS

Clinical characteristics of ALS assessment include, the ALSFRS-R score, and calculated progression rate. The ALSFRS-R is designed to quantify various indices, including bulbar function, limb motor function, and respiratory function. It facilitates dynamic monitoring and longitudinal evaluation of functional deterioration in individuals who received an ALS diagnosis. It measures physical functional capacity concerning the execution of everyday activities. Finally, progression rate was calculated using: Forty-eight minus ALSFRS-R score divided by disease duration (Rooney et al., 2017).

### 2.3 MRI acquisition parameters

These participants received cranial imaging using a GE 3.0 T MRI scanner, fitted with a dedicated multichannel head coil (48 elements). We acquired structural images using MRI with the BRAVO pulse sequence technology, specifically a T1-weighted volumetric three-dimensional fast spoiled gradient-recalled echo (3D T1W FSPGR) sequence with the specified parameters: repetition time (TR) = 9 ms, echo time (TE) = 3.4 ms, flip angle (FA) = 15°, inversion time (TI) = 450 ms, slice thickness = 1 mm, and field of view (FOV) = 224 × 224 mm^2^. Neuroimaging data were acquired utilizing a gradient-recalled echo planar imaging (GRE-EPI) protocol that is sensitive to blood oxygenation level-dependent (BOLD) contrast. The subsequent imaging specifications are as follows: TR = 2000 ms, TE = 30 ms, FA = 60°, slice thickness = 3.5 mm (comprising 36 interleaved slices for comprehensive whole-brain coverage), FOV = 224 × 224 mm^2^. The diffusion tensor imaging (DTI) protocol was executed utilizing the following acquisition parameters: TR/TE = 8000/77.9 ms, FA = 90°, FOV = 224 × 224 mm^2^, matrix size = 128 × 128, slice thickness = 2 mm, and voxel size = 1.8 mm × 1.8 mm × 2 mm.

The acquisition protocol utilized 64 diffusion-encoding directions, systematically distributed throughout three-dimensional space, with a diffusion weighting of b = 1,000 s/mm^2^ to ensure optimal diffusion weighting.

### 2.4 Image processing

The rs-fMRI data underwent comprehensive preprocessing procedures utilizing the Data Processing & Analysis of Brain Imaging software package (version 6.0; Yan et al., 2016)^1^ based on the Statistical Parametric Mapping 12 (SPM12; Friston et al., 1994).^2^ Equilibrium of the magnetization signal was established by removing the first 10 volumes. The subsequent 230 functional volumes were adjusted to account for slice-timing discrepancies and spatially realigned to address inter-scan motion. Processing included spatial smoothing (4-mm FWHM Gaussian kernel), removal of linear temporal trends, and application of a 0.01–0.1 Hz band-pass filter. Nuisance regression was deliberately excluded for gBOLD, CSF signals, and movement parameters, as they represent the primary emphasis of the present study (Han et al., 2021; Wang et al., 2023; Han et al., 2021).

**Figure 1:**
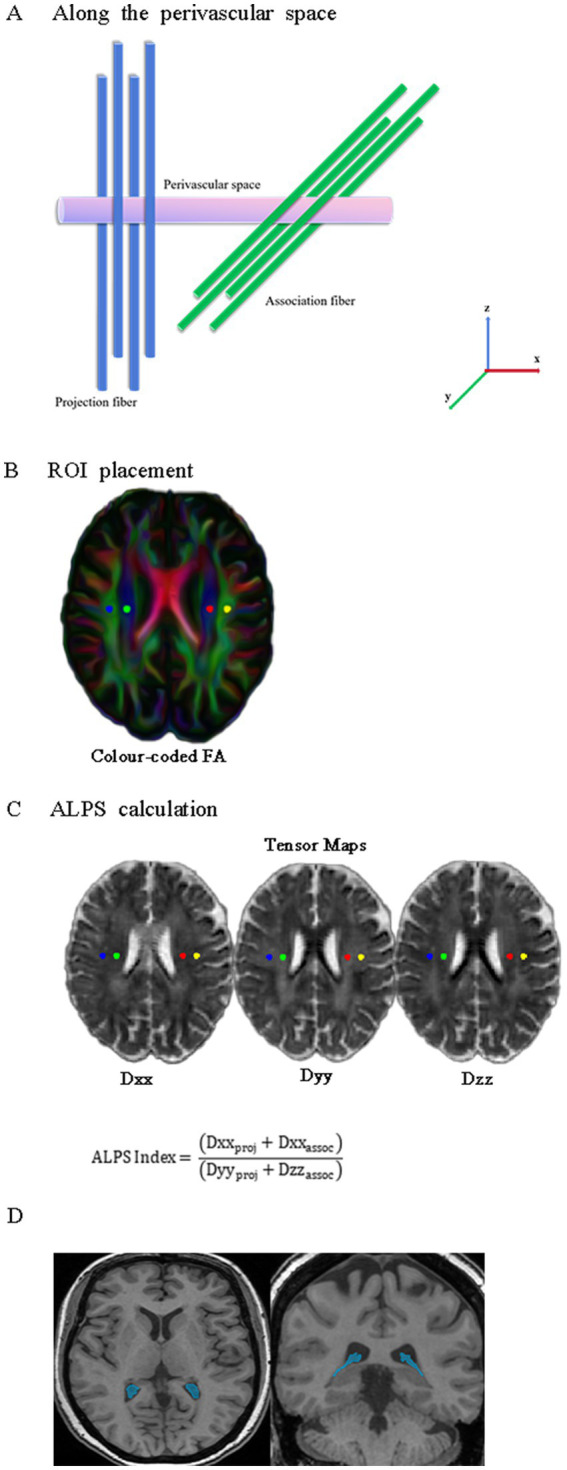
Schematic representations of DTI-ALPS and CPV. **(A)** The direction of the perivascular space (purple cylinder) is perpendicular to both the projection fibers (blue cylinder) and association fibers (green cylinder). **(B)** Four spherical ROIs, each with a 5-mm diameter, are drawn over the projection and association fibers on the FA-color map. **(C)** Calculation process for the DTI-ALPS index. Diffusivities along the Dxx (x-axis), Dyy (y-axis), and Dzz (z-axis) for projecting and associative fibers were extracted from diffusion tensor images in standard space. **(D)** Representative axial and coronal 3D T1-weighted MRI with segmentation masks of CPV (blue). DTI-ALPS, diffusion tensor imaging along the perivascular space; CPV, choroid plexus volume; ROIs, regions of interest; FA, fractional anisotropy.

For preprocessing DTI data, DTI data underwent pre-processing within FSL (FMRIB Software Library).^3^ Key steps included: (1) extracting the b0 image (topup), (2) performing brain extraction (bet; Smith, 2002), and (3) correcting for head motion and eddy currents (eddy_correct; Andersson and Sotiropoulos, 2015). Subsequently, the dtifit function was employed on each subject’s pre-processed data to generate anisotropy fraction (FA) maps. On the FA-color maps, spherical regions of interest (ROIs) with a 5-mm diameter were drawn in the areas of the projection fibers and association fibers at the level of the lateral ventricle body in both hemispheres, respectively. Dxx (x-axis), Dyy (y-axis), and Dzz (z-axis) yield diffusion parameter distributions in both individual and standardized spatial domains (Figure 1).

Automated segmentation of the structural 3D T1-FSPGR images was performed using FreeSurfer (v7.1.1).^4^ The FreeSurfer Aseg Atlas was nonlinearly registered to the individual T1 image via the recon-all pipeline. Volumes were subsequently extracted from the choroid plexus (CP) regions of interest (ROIs), a method validated for reliable CP segmentation in prior studies (Zhou et al., 2015; Egorova et al., 2019; Alisch et al., 2021). After carefully reviewing all the choroid plexus (CP) regions of interest (ROIs), we identified and manually corrected any abnormal areas to ensure accuracy. All CP ROIs underwent visual inspection and manual correction as necessary (Figure 1D).

### 2.5 Quantification of DTI-ALPS

To standardize participant data, the tensor images were co-registered to the MNI152 template space, with registration accuracy confirmed through visual inspection. The ALPS index can be expressed using the following formula (Liu et al., 2023):


$$\text{ALPS Index}=\frac{\left({\mathrm{Dxx}}_{\text{proj}}+{\mathrm{Dxx}}_{\text{assoc}}\right)}{\left({\mathrm{Dyy}}_{\text{proj}}+{\mathrm{Dzz}}_{\text{assoc}}\right)}$$

Dxxproj: Mean x-axis diffusivity in projection fiber regions; Dxxassoc: Mean x-axis diffusivity in association fiber regions; Dyyproj: Mean y-axis diffusivity in projection fiber regions; Dzzassoc: Mean z-axis diffusivity in association fiber regions.

### 2.6 Coupling between BOLD signal and CSF flow

To better assess gBOLD-CSF coupling, we first performed linear registration of the T1-weighted images to the resting-state functional MRI (rs-fMRI) data, followed by spatial normalization to the standardized MNI152 template space. The cerebral cortical gray matter areas were delineated based on the anatomical parcellation scheme provided by the Harvard-Oxford cortical atlas, which provided a consistent and reliable method for accurately pinpointing particular areas of the cortex for further examination (Desikan et al., 2006). To improve sensitivity to the CSF influx effect, our study followed an established protocol by acquiring fMRI signals from slices at the base of the cerebellum, a method that aligns with prior neuroimaging research to maintain consistency (Fultz et al., 2019; Wang et al., 2023). Gray matter BOLD signals were extracted in native space using inverse-transformed Harvard-Oxford cortical atlas ROIs.

gBOLD-CSF coupling coefficients were assessed by calculating Pearson’s r (with a lag range ±10 s) between resting-state fMRI signals from gray matter of the cortex and cerebrospinal fluid compartments. The positive crest at −4 s and the negative crest at +4 s have equivalent peak amplitudes. Therefore, the absolute value of the negative correlation coefficient at the +4-s time lag point can be used as a quantitative indicator to measure the synchronization strength between gBOLD and CSF activity. Existing research has consistently demonstrated a statistically significant association between the negative rate of change in gBOLD signals and cerebrospinal fluid pulsations (Han et al., 2021; Wang et al., 2023; Han et al., 2021). The gBOLD and CSF signals were extracted using segmented ROIs, and their coupling was quantified using lag-specific Pearson correlation. To assess significance, we performed 10,000 permutations by randomly reassigning gBOLD and CSF signal pairs across subjects, generating a null distribution for statistical testing.

### 2.7 Statistical analysis

Data from the disease group and the healthy group, as well as from the bulbar-onset ALS group and the limb-onset ALS group, were compared. For quantitative data that follow a normal distribution, the Student’s t-test is used for comparison. For data that do not follow a normal distribution, the Mann–Whitney U test is used for comparison. To evaluate intra-rater reproducibility, each region of interest (ROI) was manually delineated twice by the same rater under identical conditions. gBOLD-CSF Coupling were assessed using the intraclass correlation coefficient (ICC) in SPSS. A two-way mixed-effects model with absolute agreement definition [ICC(3.1) according to Shrout and Fleiss] was applied, as this model accounts for systematic differences across repeated measurements by a fixed rater while focusing on the consistency of absolute values. Pearson correlation analysis was used to explore the correlations among various indicators, and the FDR method was applied for multiple comparisons to correct the results. The diagnostic efficacy of each index was evaluated by drawing the receiver operating characteristic (ROC) curve and calculating the area under the curve (AUC value). Based on valuable imaging indicators, a nomogram diagnostic model for ALS was established using the logistic regression method. The ROC curve was used to evaluate the discrimination of the model, the calibration curve was drawn to assess the calibration of the model, and the DCA curve was plotted to evaluate the clinical application value of the model. Statistical analyses were performed using R (version 4.4.1), IBM SPSS Statistics (v27.0), and Prism 10.0, GraphPad. A *p*-value less than 0.05 was considered statistically significant.

## 3 Results

### 3.1 Participants demographics and clinical assessment

This study enrolled 84 subjects, consisting of 41 individuals diagnosed with ALS and 43 healthy control participants. Table 1 provides a summary of their baseline demographic features and clinical evaluation results. There were no statistically significant differences between the two groups regarding age (*p* = 0.111), BMI (*p* = 0.421), education (*p* = 0.291), or gender composition (*p* = 0.996).

**Table 1:** Baseline characteristics of participants.

	ALS (*n* = 41)	HC (*n* = 43)	*p-*value
Age	58.6 ± 9.6	55.8 ± 5.8	0.111
Sex			0.996
Female	14	15	
Male	27	28	
BMI	23.4 ± 2.8	23.8 ± 2.5	0.421
Education	9 (6, 13.5)	12 (9, 12)	0.291
ALSFRS-R score	33.2 ± 6.0		
Progression rate (/month)	1.0 ± 0.7		
Disease duration (months)	20.8 ± 13.3		
Bulbar onset	9		

ALS, amyotrophic lateral sclerosis; ALSFRS-R, revised amyotrophic lateral sclerosis functional rating scale; HC, healthy controls.

### 3.2 gBOLD signal couples with CSF signal changes

**Figure 2:**
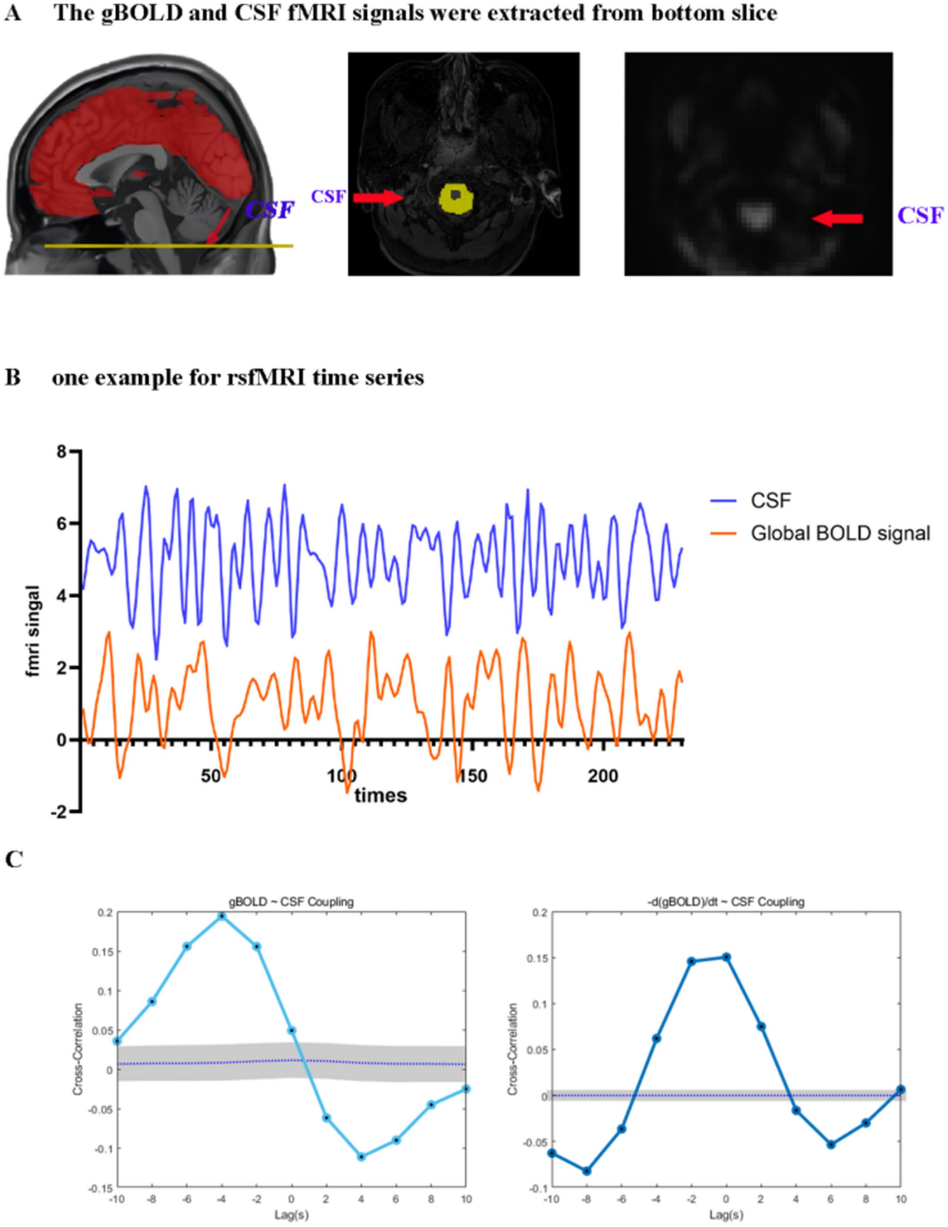
Coupling between the gBOLD signal and CSF dynamics. **(A)** The gBOLD signal was extracted across gray matter regions, while the CSF signal was extracted from the CSF regions at the bottom slice of the fMRI acquisition. **(B)** Representative gBOLD and CSF signals from a healthy control, showing corresponding changes of large amplitude. **(C)** The mean gBOLD–CSF cross-correlation function for 84 participants. The mean gBOLD–CSF coupling index exhibited a positive peak at a lag of − 4 s (B = 0.194, *p* < 0.001; permutation test, n = 10,000) and a significant negative peak at a lag of + 4 s (B = −0.111, p < 0.001; permutation test, n = 10,000). A markedly positive association (B = 0.150, p < 0.001; permutation test, n = 10,000) was observed between the time derivative of the gBOLD, denoted as -d/dt BOLD, and fluctuations in the CSF signal under zero-lag conditions. Gray bands denote 95% confidence intervals derived from signal-shuffling procedures. Both the gBOLD-CSF cross-correlation and the coupling between the gBOLD derivative and CSF signal demonstrate systematic interactions between global brain activity and CSF dynamics. gBOLD, global blood oxygen level-dependent; CSF, cerebrospinal fluid; fMRI, functional magnetic resonance imaging.

To analyze the gBOLD-CSF coupling, we acquired signals from cortical gray matter and analyzed the correlations among them. Figure 2 illustrates a clear temporal relationship between these signals, characterized by CSF signal peaks preceding gBOLD signal peaks.

Significant coupling was observed between global gray matter BOLD (gBOLD) and cerebrospinal fluid (CSF) signals. Significant positive cross-correlation was identified at an offset of −4 s (B = 0.194, *p* < 0.001; permutation test, *n* = 10,000), and the signal was accompanied by a notable negative deflection peaking at a lag of 4 s post-stimulus (B = −0.111, *p* < 0.001). Furthermore, a markedly positive association (B = 0.150, *p* < 0.001) was observed between the time derivative of the gBOLD, denoted as -d/dt BOLD (Han et al., 2021; Wang et al., 2023), and fluctuations in the CSF signal under zero-lag conditions. These findings align with existing literature, confirming a temporally structured relationship between gBOLD and CSF signals (Figure 2). Reliability measurements of gBOLD-CSF coupling demonstrated excellent consistency (ICC = 0.955).

### 3.3 gBOLD-CSF coupling, DTI-ALPS, and CPV fraction

**Figure 3:**
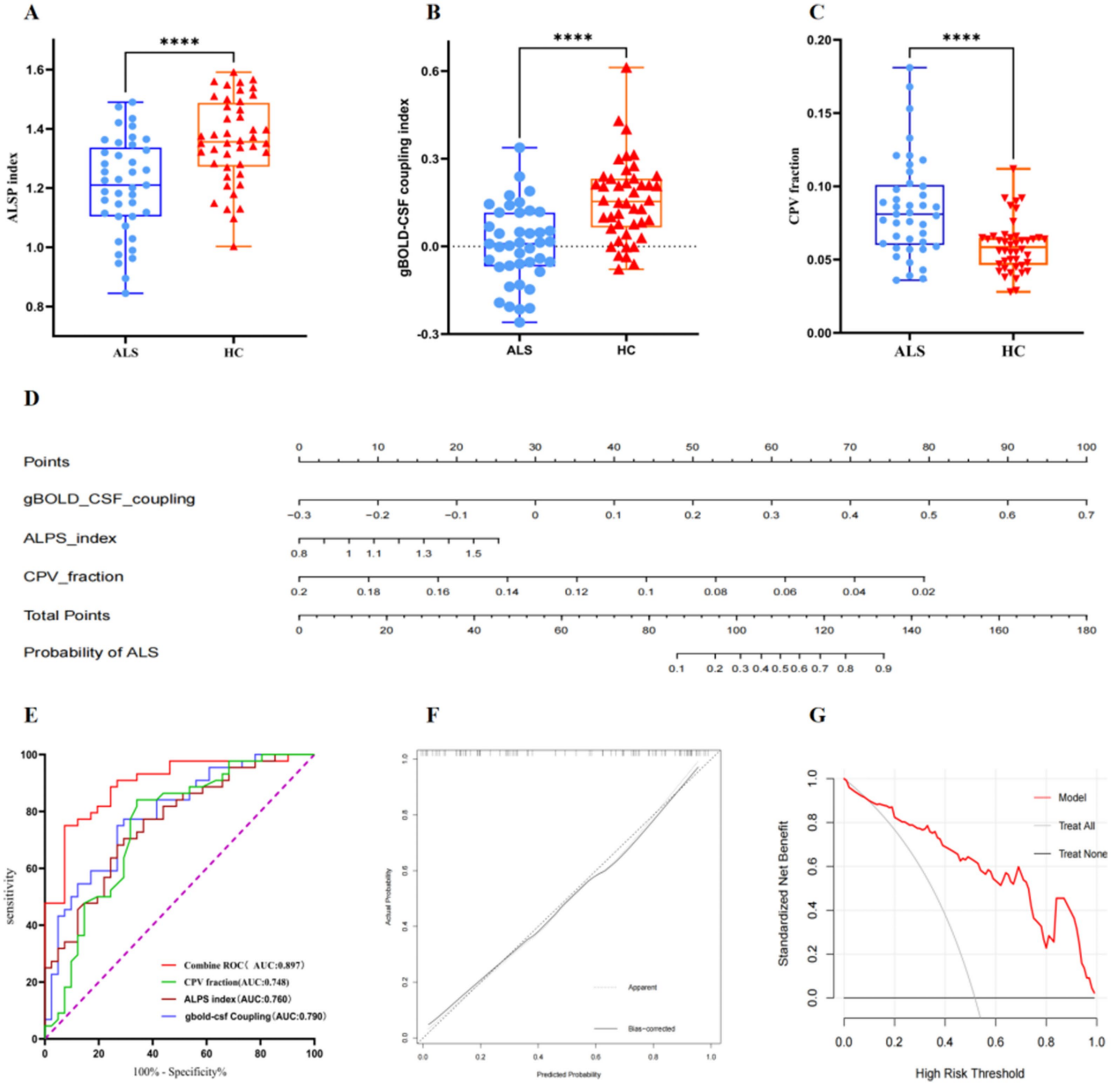
Boxplots comparing diffusivity along the ALPS index **(A)**, gBOLD–CSF coupling index **(B)**, and between CPV fraction **(C)** in patients with ALS and the HC group. **(D)** A nomogram prediction model for diagnosing ALS was developed and evaluated in ALS patients and healthy controls. **(E)** ROC curves analysis showing the diagnostic performances of ALPS index, gBOLD–CSF coupling, CPV fraction, and combined ROC in differentiating patients with amyotrophic lateral sclerosis from healthy controls. **(F)** Calibration curve for nomogram by bootstrap with 1,000 repetitions. **(G)** DCA of the nomogram. ALPS, diffusivity along the perivascular space; CPV, choroid plexus volume; gBOLD, global blood oxygen level-dependent; CSF, cerebrospinal fluid; ALS, amyotrophic lateral sclerosis; HC, healthy controls; ROC, Receiver Operating Characteristic; DCA, Decision Curve Analysis.

Statistically significant contrasts were identified in gBOLD-CSF coupling degree, DTI-ALPS values, and CPV fractions when comparing ALS patients with healthy individuals (Figure 3; Table 2). The average ALPS index was significantly reduced in ALS patients relative to the HC group (*p* < 0.001). The results of the statistical analysis indicated a significantly decreased gBOLD-CSF coupling index in individuals with ALS relative to HC subjects (*p* < 0.001). Conversely, patients with ALS exhibit significantly higher CPV levels compared to HC (*p* < 0.001). However, TIV did not differ significantly between HC and ALS patients (*p* = 0.832). The CPV fraction of ALS patients is significantly higher than that of HC (*p* < 0.001). The gBOLD-CSF coupling patterns, DTI-ALPS metrics, and CPV measurements showed comparable results between bulbar-onset and limb-onset cases. Statistical tests confirmed the absence of significant intergroup disparities (all *p* > 0.05).

**Table 2:** Parameters table of the glymphatic system in participants.

	ALS (*n* = 41)	HC (*n* = 43)	*p-value
ALPS index	1.20 ± 0.16	1.36 ± 0.14	*<0.001*
CPV (ml)	1.23 (0.99,1.59)	0.88 (0.77,1.10)	*<0.001*
TIV (ml)	1,598 ± 174	1,605 ± 148	0.832
CPV fraction (%)	0.09 ± 0.03%	0.06 (0.05,0.07)%	*<0.001*
gBOLD-CSF Coupling	0.01 ± 0.13	0.16 ± 0.14	*<0.001*

ALPS, diffusivity and the index along the perivascular space; ALS, amyotrophic lateral sclerosis; CPV, choroid plexus volume; HC, healthy controls; TIV, total intracranial volume; gBOLD-CSF Coupling, the coupling strength of global blood-oxygen-level-dependent (gBOLD) signals and cerebrospinal fluid (CSF); *Bonferroni correction is used for multiple testing adjustments, and statistical significance can be achieved if *p* < 0.05/5.

### 3.4 Diagnostic performances of the gBOLD-CSF coupling, DTI-ALPS, and CPV fraction

To evaluate the performance of various biomarkers in terms of diagnostic accuracy for amyotrophic lateral sclerosis, we constructed ROC curves for each indicator and computed the corresponding AUC values. The parameters are as follows: gBOLD-CSF Coupling (AUC:0.790, 95% CI:0.694–0.885, cut-off point:0.072, specificity:0.732, sensitivity:0.750), DTI-ALPS (AUC:0.760, 95%CI:0.660–0.881, cut-off point:1.321, specificity:0.732, sensitivity:0.682) and CPV fraction (AUC:0.748, 95%CI:0.641–0.885, cut-off point:0.068, specificity:0.659, sensitivity:0.841). The AUC values for the above parameters are all above 0.70. The ROC analysis of the combined model, incorporating gBOLD-CSF coupling, DTI-ALPS, and CPV scores, shows an AUC of 0.897 (95% CI: 0.830–0.963, cut-off point:0.690, specificity:0.927, sensitivity:0.750), confirming high diagnostic reliability (Figure 3E). We developed a nomogram diagnostic model (Figure 3D). The ROC curve was plotted to evaluate the model’s performance, with an AUC of 0.897 (95% CI: 0.830–0.964; Figure 3E). The calibration curve illustrates a great concordance between the model’s predictions and the actual observations (Figure 3F). DCA indicates that when the threshold probability is greater than 15%, the application of the model yields more benefits (Figure 3G).

### 3.5 Correlation analysis between imaging indicators and clinical scales as well as among various indicators

**Figure 4:**
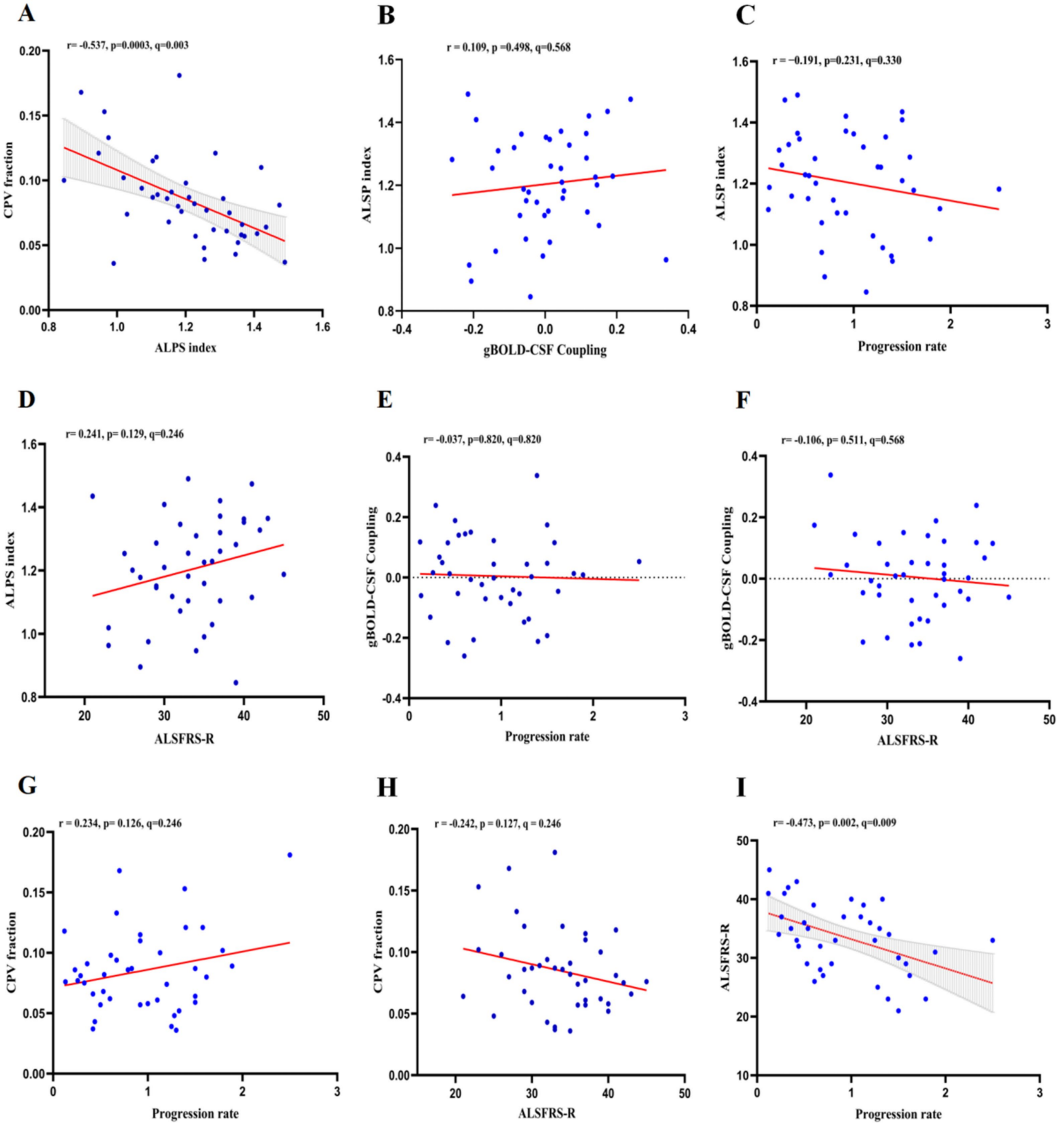
In ALS patients, the associations between ALPS index, gBOLD-CSF coupling, CPV fraction, and ALSFRS-R score and progression rate were as follows: **(A)** ALPS index was negatively correlated with CPV fraction (r = −0.537, *p* = 0.0003, q = 0.003); **(B)** ALPS index was not significantly correlated with gBOLD-CSF coupling (r = 0.109, *p* = 0.498, q = 0.568); **(C)** ALPS index was not significantly correlated with progression rate (r = −0.191, *p* = 0.231, q = 0.330); **(D)** ALPS index was not significantly correlated with ALSFRS-R score (r = 0.241, *p* = 0.129, q = 0.246); **(E)** gBOLD-CSF coupling was not significantly correlated with progression rate (r = −0.037, *p* = 0.820, q = 0.820); **(F)** gBOLD-CSF coupling was not significantly correlated with ALSFRS-R score (r = −0.106, *p* = 0.511, q = 0.568); **(G)** CPV fraction was not significantly correlated with progression rate (r = 0.234, *p* = 0.126, q = 0.246); **(H)** CPV fraction not significantly correlated with ALSFRS-R score (r = −0.242, *p* = 0.127, q = 0.246); **(I)** ALSFRS-R score was negatively correlated with progression rate (r = −0.473, *p* = 0.002, q = 0.009).

ALPS, diffusivity along the perivascular space; gBOLD–CSF, global blood-oxygen-level-dependent (BOLD) signals and cerebrospinal fluid (CSF); CPV, choroid plexus volume; ALSFRS-R, Revised ALS Functional Rating Scale.

The study population demonstrated an inverse correlation between the ALPS and CPV proportions (*r* = −0.537, *p* = 0.0003, q = 0.003) and a statistically significant negative association between the ALSFRS-R score and the rate of disease progression (*r* = −0.473, *p* = 0.002, q = 0.009; Figure 4). The ALPS index showed no significant correlation with gBOLD-CSF coupling (*r* = 0.109, *p* = 0.498, q = 0.568), progression rate (*r* = −0.191, *p* = 0.231, q = 0.330), or ALSFRS-R score (*r* = 0.241, *p* = 0.129, q = 0.246). Similarly, gBOLD-CSF coupling was not significantly correlated with progression rate (*r* = −0.037, *p* = 0.820, q = 0.820) or ALSFRS-R score (*r* = −0.106, *p* = 0.511, q = 0.568). Additionally, the CPV fraction did not show significant correlations with progression rate (*r* = 0.234, *p* = 0.126, q = 0.246) or ALSFRS-R score (*r* = −0.242, *p* = 0.127, q = 0.246). To assess whether the disease course has an impact on various indicators, we conducted a correlation analysis between the disease course and each indicator. The disease course was only significantly correlated with the disease progression rate (*r* = −0.696, *p* < 0.001; Supplementary Table S1). To explore whether the correlation between the ALPS index and the CPV score is affected by the disease duration, we re-conducted the correlation analysis with the disease duration as a covariate. The results showed that the ALPS index was still significantly correlated with the CPV score (*r* = −0.541, *p* < 0.001).

## 4 Discussion

This study assessed lymphatic system function in ALS patients by utilizing three MRI-based markers: gBOLD-CSF coupling, DTI-ALPS index, and CPV fraction. This study pioneers the application of gBOLD-CSF coupling in ALS, combined with DTI-ALPS index and CPV fraction analyses, and further explored an association between the disease and the lymphatic system. Under normal physiological conditions, factors like intracranial pressure, cranial cavity volume, total cerebrospinal fluid volume, cerebral blood flow, and interstitial fluid volume remain in balance. Decreases in global brain activity lead to reduced cerebral blood volume and pressure, followed by increased CSF inflow. Resting-state gBOLD-CSF coupling may reflect neural and physiological processes associated with the glymphatic clearance mechanism. The gBOLD signal shows sleep-dependent behavior similar to the lymphatic system (Fukunaga et al., 2006). Sleep deprivation enhances the gBOLD signal (Yeo et al., 2015), while caffeine reduces it (Wong et al., 2013). Brain imaging data reveal that fluctuations in the 0.01–0.1 Hz range of gBOLD signals may reflect underlying slow vascular pulsations. These pulsations are implicated in driving glymphatic CSF flow (Iliff et al., 2013; Kiviniemi et al., 2015; Mestre et al., 2018), and may be mediated by the autonomic system during transient arousal changes reflected in large gBOLD peaks (Ozbay et al., 2018). Spontaneous low-frequency modulations in vessel tone are directly linked to perivascular clearance (van Veluw et al., 2020). Low-frequency rs-fMRI data indicate that the gBOLD-CSF coupling likely represents a synchronized neurophysiological process tightly associated with the glymphatic clearance mechanism (Han et al., 2021; Han et al., 2021). Therefore, changes in the coordinated activity between the gBOLD fluctuations and the CSF inflow can be measured by analyzing the correlation between the two signals. Last, strong CSF movements were indeed found to be coupled with the large gBOLD signal, as confirmed in the present study, adding a new piece of evidence for the glymphatic system in ALS. In our research, ALS causes a significantly decreased gBOLD-CSF coupling compared to healthy individuals. The decreased ALPS parameters in ALS patients confirm existing evidence of diminished glymphatic activity (Liu et al., 2023). The HC group showed a lower CPV fraction, whereas ALS patients displayed a significantly increased CPV fraction.

gBOLD-CSF coupling assesses the synchrony between neural activity and brain/CSF motion by integrating the whole-brain BOLD signal with CSF movement, thereby reflecting the function of the glymphatic system. Studies have shown that gBOLD signals during sleep are significantly associated with CSF movement, which is an important component of the lymphatic system. Therefore, resting-state global activity and its physiological regulation are considered closely related to the lymphatic clearance mechanism (Han et al., 2021). The DTI-Alps method uses diffusion tensor imaging to calculate the ratio of directional diffusion in the perivascular space (PVS) to interstitial water molecule diffusion. A low ALPS index indicates restricted water molecule flow in the PVS and can be used to assess the function of the glymphatic system (Taoka et al., 2024). The CPV produces cerebrospinal fluid, forms the blood-cerebrospinal fluid barrier, and removes toxic wastes and metabolites from the central nervous system (Christensen et al., 2022). These three methods assess the function of the glymphatic system from different perspectives, providing a more comprehensive evaluation.

Previous studies and our current experiment have demonstrated a correlation between the DTI-ALPS index and CPV fraction (Choi et al., 2025). Therefore, we further explored the correlation between gBOLD-CSF coupling and the DTI-ALPS index and CPV fraction, and found no significant correlation. This might be because the physiological mechanisms reflected by gBOLD-CSF coupling and DTI-ALPS and CPV fractions differ in pathological basis, disease development stage or measurement methods, which leads to no significant correlation between them. The specific reasons need further verification. After adjusting for the potential confounding factor - disease duration, the ALPS index remained significantly correlated with the CPV score. This result suggests that both may be closely related to the pathological state of the disease. Further research can be conducted in the future to explore their correlation and underlying mechanisms in depth. The relationship between the ALSFRS-R score and the disease progression rate indicates that delta FS (ΔFS) is a simple and sensitive clinical prognostic biomarker that can more intuitively reflect the degree of disease progression (Labra et al., 2016; Ludolph et al., 2024). When initially exploring the correlations, it was found that disease progression was correlated with both the ALSFRS-R score and disease duration. Since the disease progression indicator itself is calculated based on the ALSFRS-R score and disease duration, the correlations between it and the two are expected. Under such circumstances, further analysis of their correlations has limited clinical significance.

No significant correlation was found between imaging indicators, such as gBOLD-CSF coupling, DTI-ALPS, and CPV fraction, and clinical scores in ALS patients. Similar to the results of a recent study, a reduction in the DTI-ALPS index was observed in ALS patients, but there was no significant correlation with clinical parameters (Sharkey et al., 2024). Although imaging markers like gBOLD-CSF coupling do not strongly correlate with clinical scores, they remain promising biomarkers. These markers may be more sensitive in early disease stages and can aid preclinical diagnosis and progression monitoring, rather than only reflecting current disease status. The ALSFRS-R scale is limited by floor and ceiling effects (Hayden et al., 2022). In the early stage of the disease, the brain can maintain its function through compensatory mechanisms, keeping some clinical symptoms normal (Blesa et al., 2017), while imaging examinations can already detect abnormalities in the brain at this time. This non-linear change may make it difficult for cross-sectional studies to discover linear relationships. Compared with clinical scores, imaging indicators such as gBOLD-CSF coupling can provide more objective and sensitive early information. Therefore, future research should focus on disease staging and longitudinal studies to further validate the relevant findings.

Notably, we combined gBOLD-CSF coupling, DTI-ALPS index and CPV fraction to establish a nomogram model (AUC: 0.897), demonstrating the application value of multi-index methods in evaluating lymphatic system changes in ALS. Neuroimaging data are more objective than clinical scores and less affected by human bias, providing a new perspective for ALS research. The study deepened the understanding of the pathogenesis of ALS, proposed new indicators that are helpful for early diagnosis, and is expected to support clinical decision-making. However, due to the limited sample size, the current model is still preliminary and lacks a validation set. In the future, a larger sample will be included to build a more complete model.

Therefore, our research has confirmed that patients with ALS will experience damage to the function of the glymphatic system. This research has several limitations that need to be recognized. First, the survey was conducted in a formal setting, which might, to some extent, affect the generalizability of the research results. Second, the limited sample size precluded a precise evaluation of the effect of disease progression on glymphatic system dysfunction. Third, while the relationship between DTI-ALPS and gBOLD-CSF coupling has been confirmed by multiple studies and is frequently used to assess the glymphatic system’s CSF clearance function, current measurements remain indirect due to the structural and functional complexity of this system. Therefore, future research should conduct multi-center replication experiments, longitudinal cohort tracking, and animal experiments to systematically and comprehensively explain the specific physiological mechanisms and clinical application values of these neuroimaging markers.

## 5 Conclusion

Patients with amyotrophic lateral sclerosis (ALS) have damage to the structure and function of the lymphatic system. gBOLD-CSF coupling is helpful for the diagnosis of ALS, further confirming previous studies on the DTI-ALPS index and CPV fraction. By combining gBOLD-CSF coupling, the ALPS index, and CPV fraction, a diagnostic model was developed, which has good diagnostic efficacy and clinical application value.

## Data Availability

The raw data supporting the conclusions of this article will be made available by the authors, without undue reservation.

## Footnotes

^1^ http://rfmri.org/dpabi

^2^ https://www.fil.ion.ucl.ac.uk/spm/software/spm12/

^3^ https://fsl.fmrib.ox.ac.uk/fsl/fslwiki/

^4^ http://surfer.nmr.mgh.harvard.edu
