# Supplementary material for: Glymphatic dysfunction in amyotrophic lateral sclerosis: a multimodal MRI investigation of brain-CSF functional and structural dynamics
Source: Front Neurosci. 2025 Oct 6;19:1666114. doi: 10.3389/fnins.2025.1666114 (PMC12536026; doi:10.3389/fnins.2025.1666114)
Supplement: Supplementary file 1 [file Table_1.docx]

Table S1 Correlation analysis of disease course and imaging indicators

|  |  | gBOLD-CSF Coupling | ALPS index | CPV fraction | ALSFRS-R | Progression rate |
| --- | --- | --- | --- | --- | --- | --- |
| Disease duration (months) | r | 0.057 | 0.041 | 0.049 | -0.023 | -0.696 |
|  | p | 0.722 | 0.798 | 0.761 | 0.885 | <0.001 |
| ALPS, diffusivity along the perivascular space; gBOLD–CSF, global blood-oxygen-level-dependent (BOLD) signals and cerebrospinal fluid(CSF);CPV, choroid plexus volume;ALSFRS-R, revised amyotrophic lateral sclerosis functional rating scale. | | | | | | |
